# Prevalence of bovine endometritis, virulence mechanisms of *Escherichia coli*, and current therapeutic strategies: A review

**DOI:** 10.1080/21505594.2026.2707822

**Published:** 2026-07-28

**Authors:** Mengsen Wang, Xiangfu Wen, Xiaohan Li, Hao Li, Jia Cheng, Mingchao Liu

**Affiliations:** College of Veterinary Medicine, Hebei Agricultural University, Baoding, China

**Keywords:** Bovine endometritis, Escherichia coli, virulence factors, pathogenesis, therapeutic methods

## Abstract

Dairy cow endometritis is a common postpartum disease influenced by geographic, management, environmental, diagnostic, and socio-economic factors. *Escherichia coli* (*E. coli*) was prioritized for its early colonization, frequent detection, and key inflammatory role in endometritis. Its pathogenicity depends on virulence factors, including adhesins, toxins, lipopolysaccharides (LPS), capsule antigens, siderophores, and biofilm formation. These factors jointly promote bacterial colonization, immune activation, tissue damage, and disease progression. This study combines a systematic review and meta-analysis to assess the prevalence and distribution of *E. coli*, and provides a descriptive synthesis of its key virulence factors, pathogenic mechanisms, and treatment strategies. Unlike descriptive reviews, this study links epidemiological patterns with pathogenic mechanisms and therapies. Main limitations include high epidemiological heterogeneity and limited clinical validation of therapeutic strategies. It further evaluates current treatment strategies to assess the feasibility of novel therapies, aiming to provide a reference for precise prevention and control in clinical practice.

## Introduction

Bovine endometritis is a common postpartum disease of the reproductive system, characterized clinically by persistent inflammation of the endometrium after 21 days postpartum [1]. This disease severely affects the reproductive efficiency of dairy cows and poses a significant threat to the economic sustainability of the global dairy industry [2]. The postpartum period is a critical stage for dairy cows, during which metabolic stress, immune suppression, cervical dilation, and endometrial injury significantly increase the risk of disease [3]. Global epidemiological data indicate that postpartum uterine infections are common in dairy cows. Clinical endometritis persisting beyond the first three weeks postpartum is generally reported in approximately 15–20% of cows [1]. Under high-risk conditions, postpartum uterine diseases (including metritis and endometritis) may affect up to 40% of dairy cows, especially in high-producing cows experiencing increased metabolic load and periparturient stress [4]. In high-altitude tropical regions, environmental and climatic stressors may further compromise reproductive health and increase the risk of postpartum uterine diseases [5].

The pathogenesis of bovine endometritis is multifactorial, involving invasion by pathogenic microorganisms, imbalance in host immune-metabolic regulation, and inadequate environmental and management conditions [6]. Bacterial infection is the main cause of endometritis in dairy cows, with *E. coli* being a major pathogen due to its early colonization ability and key pathogenic role, although it is not the only causative agent [7]. Studies have shown that endometrial pathogenic *E. coli* can induce host inflammatory responses and tissue damage through multiple virulence-associated mechanisms, including lipopolysaccharides (LPS)-mediated immune activation, adhesion and invasion of endometrial cells, toxin production, iron acquisition systems, and other pathogenicity-associated factors [1,8]. In addition, the biofilm-forming ability and antimicrobial resistance of *E. coli* can enhance its capacity for persistent infection, leading to clinical treatment failure and disease recurrence, and driving research on alternative therapeutic strategies [9,10]. Studies have shown that when bacterial load exceeds a critical threshold, it triggers an excessive inflammatory response, leading to the development of clinical uterine disease [2,11,12]. The pathogenicity of the bacteria depends on the synergistic action of a series of virulence factors, including type 1 fimbrial adhesin (FimH) and P fimbrial adhesin (PapG) for bacterial colonization [13,14], hemolysin A (HlyA), cytotoxic necrotizing factor 1 (CNF1), and LPS for tissue damage and induction of inflammatory responses [15–17], K1 antigen and O antigen for immune evasion [17,18], siderophore for nutrient acquisition [19], and biofilm formation for persistent infection [20]. More broadly, infectious agents can impair female reproductive function through immune-mediated inflammatory mechanisms, thereby disturbing the reproductive microenvironment and fertility-related outcomes [21]. These factors together form a complete pathogenic network, ultimately leading to structural damage of the endometrium, functional impairment, and reduced reproductive performance.

At present, antibiotics remain the main method for treating bovine endometritis [22]. However, the widespread use of antibiotics, especially irrational application, has led to increasingly serious public health and livestock safety issues, including enhanced antimicrobial resistance, drug residues in dairy and meat products, and imbalances in the reproductive tract microbiota [23,24]. Therefore, developing safe, effective, and sustainable alternative therapies is particularly urgent.

Systematic analysis of the epidemiology, clarification of pathogenic mechanisms, and development of effective prevention and control strategies are essential to reduce economic losses from endometritis and mitigate antibiotic resistance [25]. Although previous studies have explored postpartum uterine diseases in dairy cows, reproductive tract microbiota, and treatment interventions, existing evidence mostly focuses on individual aspects of the disease, such as diagnosis, specific pathogens, or treatment [1,26–28]. To our knowledge, there is currently still a lack of comprehensive integrated research that can link global epidemiological patterns, regional differences, *E. coli* virulence factors, pathogenic mechanisms, and existing and emerging prevention and control strategies. To address this gap, this review aims to describe the reported global incidence and spatial distribution of bovine endometritis, compare regional differences and potential contributing factors, identify the major virulence factors of *E. coli* and their pathogenic roles in endometritis, and evaluate current prevention and control strategies for this disease.

## Prevalence and spatial distribution of bovine endometritis

The literature search strategy includes the databases searched (PubMed and Web of Science), the search time range (January 2015 to December 2025), and the combination of keywords used for study identification (such as “cow,” “dairy cow,” “endometritis,” “clinical endometritis,” “subclinical endometritis,” “prevalence,” and “epidemiology”). The study selection criteria are also clarified: only studies that report both the number of endometritis cases in cows and the total number of animals examined were included, in order to extract or calculate prevalence; original research meeting peer review requirements was considered valid data, whereas review articles, conference abstracts, case reports, and studies lacking sufficient epidemiological information were excluded.

**Figure 1.**
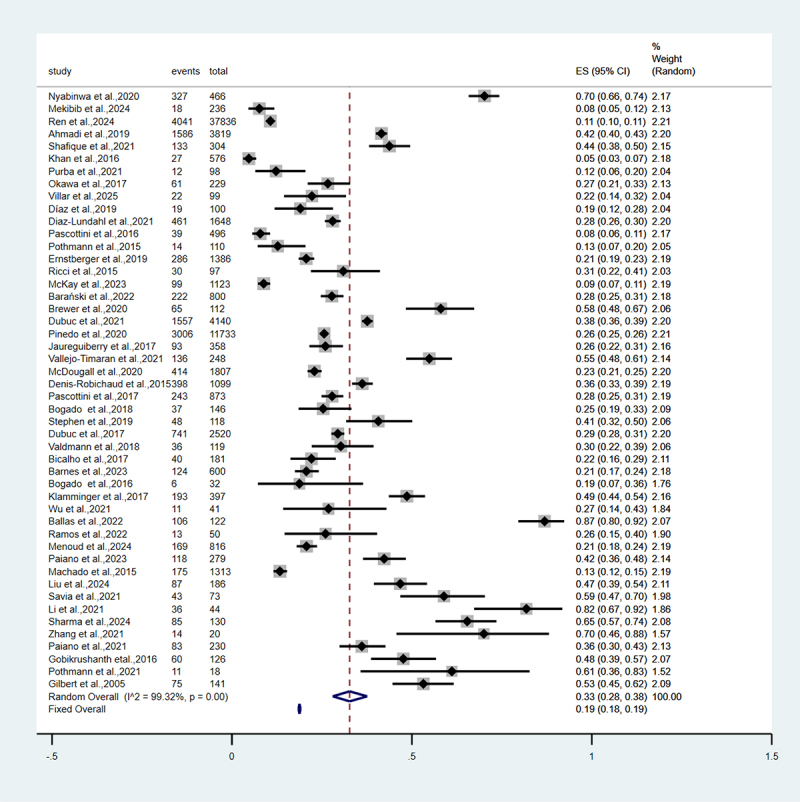
Forest plot of the meta analysis of the prevalence of endometritis in dairy cows worldwide. This figure presents the results of a random effects model meta analysis based on studies from the past 10 years. The “total” and “events” columns correspond to the total number of animals in each study and the number of cases positive for endometritis, respectively. Each study is represented by a horizontal line showing its effect size (prevalence) and 95% confidence interval (CI), with the size of the square reflecting its weight. On the right, the pooled prevalence and 95% CI of each study and the overall combined estimate are summarized. The diamond represents the pooled effect size, with its width corresponding to the 95% CI of the pooled effect. The heterogeneity assessment results are I^2^ = 99.32%, *p* < 0.001.

This research integrates epidemiological data from nearly 50 countries and regions worldwide over the past decade through a meta analysis approach, aiming to systematically assess the overall incidence and heterogeneity characteristics of bovine endometritis (Figure 1). Statistical analysis showed significant heterogeneity among the included studies (I^2^ = 99.32%, *p* < 0.001). Therefore, using the appropriately applicable random-effects model, the estimated true global prevalence of endometritis in cows is 33% (95% CI: 0.28–0.38). The fixed-effects model’s significantly lower estimate of 19% (95% CI: 0.18–0.19) demonstrates the result of ignoring geographical and management variability. This difference indicates that in practical prevention and control work, it is necessary to fully consider the impact of factors such as geographical location and farming management.

**Figure 2.**
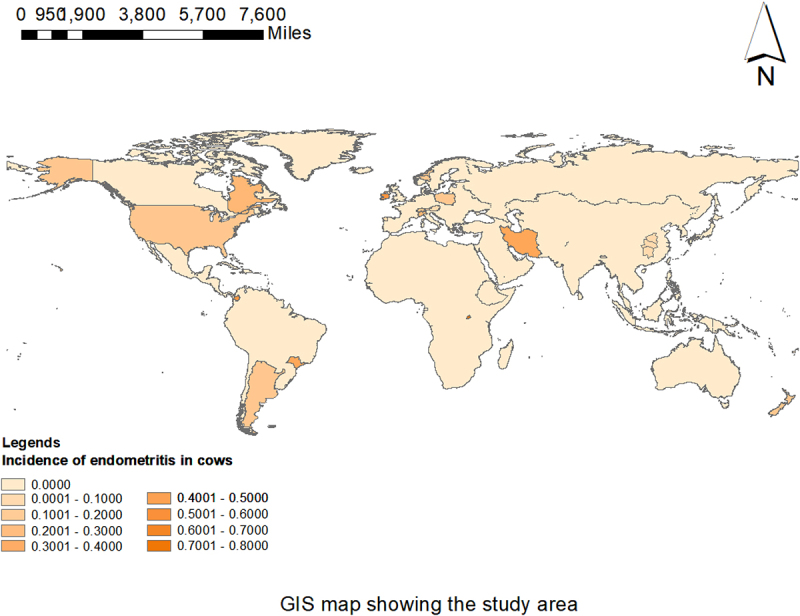
Spatial distribution of bovine endometritis prevalence worldwide. This figure is based on epidemiological data from nearly 50 countries and regions over the past 10 years and was created using ArcMap 10.8 software. The map uses color gradients to represent differences in prevalence, ranging from yellow (low prevalence) to red (high prevalence), intuitively illustrating the global heterogeneity and regional clustering of the disease. The specific prevalence rates incorporated are as follows: Africa shows a striking contrast between Rwanda (70.20%) and Ethiopia (7.63%). In Asia, rates are high in Punjab, Pakistan (43.75%) and Iran (41.53%), moderate in regions of Japan (Fukuoka prefecture 26.60%; Hiroshima 12.24%) and Central China (10.68%), and low in Meghalaya, India (4.61%). Europe exhibits a gradient from high in the northwest (Ireland 58.04%; Hedmark and Trøndelag, Norway 27.97%), moderate in central/southern regions (Piedmont, Italy 31.00%; Northern Poland 27.75%; Switzerland 20.63%; Austria 12.70%), to lower rates in parts of Spain (Cantabria 22.22%; Lugo 19.00%) and Flanders, Belgium (7.86%). In the Americas, a high-prevalence belt spans South America (Antioquia, Colombia 54.84%; São Paulo state, Brazil 42.29%; Argentina 26.00%), with North America showing variation (Quebec, Canada 37.61%; United States 25.62%). New Zealand (22.90%) represents a moderate prevalence in Oceania. The base map data is sourced from natural earth.

The spatial distribution of bovine endometritis prevalence demonstrates considerable heterogeneity worldwide, as synthesized from epidemiological data across nearly 50 countries and regions (Figure 2). Africa demonstrates the most extreme disparity, where Rwanda stands as a global outlier with a prevalence of 70.20% [29], contrasting sharply with Ethiopia at 7.63% [30]. In Asia, high rates are reported in Pakistan’s Punjab (43.75%) [31] and Iran (41.53%) [32], while parts of Japan and Central China show moderate levels, and Meghalaya, India (4.61%) [33] represents a notable low prevalence area. Europe presents a northwest-to-southeast gradient, with high incidence in Ireland (58.04%) [34] and Hedmark and Trøndelag, Norway (27.97%) [35], transitioning to moderate rates in central/southern regions and lower rates in countries like Flanders, Belgium (7.86%) [36]. A contiguous high prevalence belt spans South America, including Antioquia, Colombia (54.84%) [5] and São Paulo State, Brazil (42.29%) [37], whereas North America shows internal variation, with Canada’s Quebec (37.61%) [38] exceeding the U.S. average (25.62%) [39]. New Zealand (22.90%) [40] represents a moderate-risk region in Oceania.

The observed spatial distribution heterogeneity of bovine endometritis highlights that its prevalence is influenced by multiple factors. In this meta-analysis, the variables directly included in the statistical methods were the number of positive cases, total sample size, publication year, and the geographic location of each study, which enabled the estimation of pooled prevalence and the exploration of heterogeneity (Figures 1 and 2). Other potential influencing factors, including husbandry practices, environmental conditions, regional pathogen strains, diagnostic criteria, coverage of preventive veterinary services, and socioeconomic factors, were not quantitatively included in the analysis due to limited reporting in the original studies; however, they are discussed in the article as potential contributors to the observed heterogeneity. Recent advances in diagnostic technologies may further improve disease surveillance and epidemiological assessment. For example, CRISPR/Cas12a-based assays have demonstrated rapid and sensitive detection of pathogens such as *E. coli* [41], while portable isothermal amplification methods provide practical solutions for field-based pathogen detection [42]. In addition, protein-based biomarkers are increasingly being explored as diagnostic indicators for bovine diseases, offering complementary tools for disease monitoring and early diagnosis [43]. This review utilizes global data integrated through GIS technology, providing valuable spatial epidemiological clues and references for identifying high-risk areas, comparing disease pressures under different production systems, and exploring the specific underlying driving mechanisms in the future.

## Virulence factors

The pathogenic potential of bacteria is usually closely related to their virulence factors. *E. coli* carries a variety of major virulence factors, including FimH, PapG, HlyA, CNF1, LPS, K1 antigen, and O antigen, siderophore, biofilm [44]. These virulence factors have different properties, each playing a unique role in the pathogenesis.

### FimH

**Figure 3.**
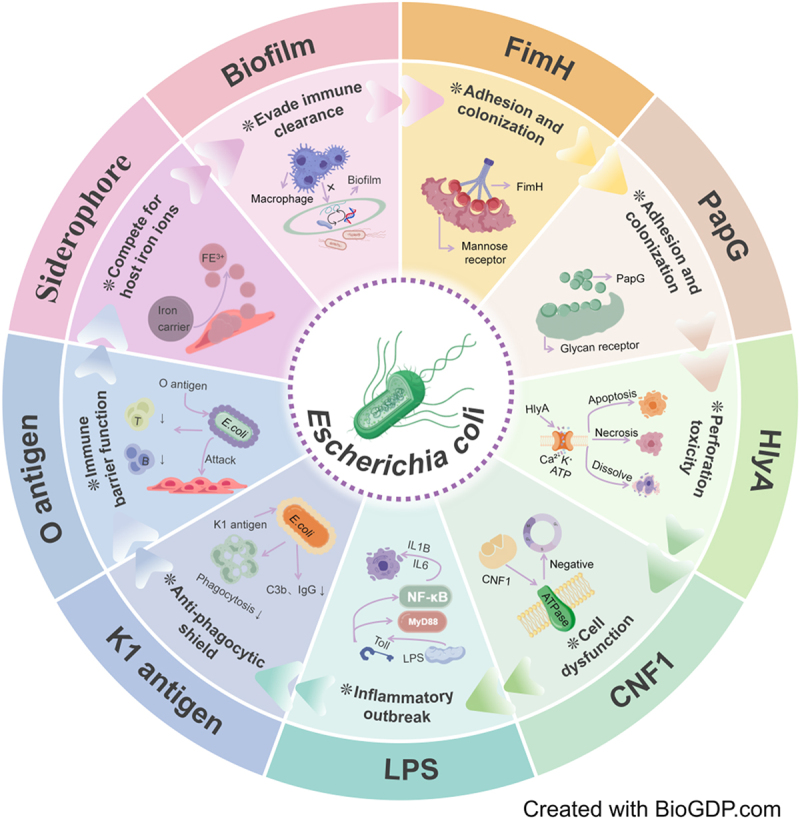
The main virulence factors of *E. coli* and their functional characteristics. The adhesins FimH and PapG mediate specific binding to receptors on endometrial epithelial cells, thereby promoting initial adhesion and colonization and contributing to activation of innate immune signaling. The toxin HlyA forms transmembrane pores, leading to ion leakage and host-cell injury/lysis, which facilitates bacterial invasion and interferes with early immune clearance. CNF1 promotes sustained activation of Rho-family GTPases, remodeling the host cytoskeleton, enhancing bacterial internalization, and amplifying inflammatory responses. LPS, a key endotoxin, activates TLR4-associated pathways and triggers NF-κB/MAPK-driven expression of inflammatory mediators and neutrophil recruitment. Surface polysaccharide structures (K1 capsular antigen and O antigen) enable immune evasion by providing a physical barrier and reducing complement-mediated opsonization. Siderophores support growth and virulence by scavenging iron under host nutritional immunity. Biofilms, composed of extracellular DNA, proteins, and polysaccharide matrix, increase resistance to host defenses and antimicrobials, thereby promoting persistent infection and recurrence.

FimH is a mannose-binding adhesin expressed by pathogenic *E. coli* and other members of the Enterobacteriaceae, located at the tip of type 1 pili, and exhibits stereochemical specificity [13,45]. Compared with other adhesins, more than 95% of clinical isolates of *E. coli* express type 1 fimbriae, indicating their prevalence in the pathogenic process [46,47]. Importantly, epidemiological studies have shown that *fimH*-positive intrauterine *E. coli* isolates are strongly associated with the occurrence of bovine endometritis [48]. FimH mediates the initial colonization of bacteria by specifically binding to mannose receptors on the surface of host cells (such as receptors on endometrial cells), which is a crucial step for bacteria to successfully establish themselves and further invade host cells [45,49] (Figure 3). In addition, other mannose-rich structures, such as mucins, may also serve as high-capacity binding substrates for FimH, promoting its adhesion, thereby contributing to biofilm formation and triggering bacteria-specific mucosal immune responses. Upon binding to mannose, FimH undergoes a conformational change to form a high-affinity, highly stable complex, enhancing the bacteria’s persistence in host tissues and promoting the formation of intracellular bacterial communities (IBCs) and biofilms [50–52]. Compared with other virulence factors, FimH-mediated initial colonization is a prerequisite for the subsequent action of virulence mechanisms.

FimH not only mediates adhesion but can also specifically recognize various receptors on the surface of host cells, including carcinoembryonic antigen-related cell adhesion molecule 6 (CEACAM6) and Toll-like receptor 4 (TLR4) [53–55]. Notably, FimH can directly bind to TLR4 and trigger a strong innate immune response by activating the MyD88-dependent signaling pathway [56,57]. MyD88, as a key adaptor protein, links TLR/IL-1 R to downstream signaling molecules through its death domain and TIR domain, thereby activating important signaling pathways such as NF-κB and MAPK [58,59]. Different TIR domain adaptor proteins (such as MyD88, TIRAP, and TRIF) mediate signaling for different TLRs in a specific manner, determining the diversity and specificity of Toll-like receptor signaling pathways [60]. Activation of MyD88 signaling initiates a series of inflammatory responses and can even induce systemic inflammatory response syndrome, while also regulating immune cell infiltration, polarization, and immune evasion in the tumor microenvironment [61,62]. During infection, FimH protein exhibits dual functions. It promotes bacterial colonization via adhesion and triggers inflammation through the TLR4 pathway, illustrating a complex host-pathogen interaction [63].

### PapG

PapG is a key adhesin protein located at the tip of the P pili of *E. coli*, serving as a core virulence factor and responsible for mediating the specific binding between the bacteria and the host glycan receptors [14,64] (Figure 3). P pili are heteropolymeric protein fibers composed of multiple subunits including PapA, PapH, PapG, PapE, PapF, and PapK, featuring a rigid stem and a flexible tip [65–67]. Although PapG constitutes only a small part of pilus assembly and its absence does not affect the overall formation of pili, it significantly weakens the bacteria’s ability to recognize and adhere to glycan receptors containing the Gal(α1–4)Gal structure (such as Forssman antigen Gb5, globotetraosylceramide Gb4, and globotriaosylceramide Gb3) [68,69]. Based on differences in receptor-binding specificity, PapG can be divided into three types, which together determine the tissue tropism and infectious capability of *E. coli*. These glycolipid receptors have been identified in bovine cells and tissues, suggesting that similar receptor-mediated adhesion mechanisms may contribute to host colonization by pathogenic *E. coli* (EnPEC) in the endometrium [70,71]. However, the specific distribution of PapG-recognized receptors in bovine endometrial tissue has not yet been fully clarified. Although direct evidence linking specific PapG variants to bovine endometritis remains limited, PapG is a well-characterized adhesin that plays a role similar to FimH in the early colonization stages, thereby ensuring the survival of *E. coli* within the host [72]. In the pathogenic mechanism, PapG binds with high affinity to Toll-like receptor 2 (TLR2), effectively activating the innate immune response. PapG can induce morphological maturation of bone marrow-derived dendritic cells (BMDCs), upregulate the expression of co-stimulatory molecules and MHC class I/II molecules, and promote the production of pro-inflammatory cytokines [73]. In addition, PapG can protect *E. coli* from neutrophil-mediated killing and promote the formation and invasion of intracellular bacterial communities [74,75]. Notably, pathogenic *E. coli* strains derived from cows with endometritis exhibit stronger invasiveness and adhesion to stromal cells, which helps enhance their early colonization and infection process in the endometrium [76,77]. Disrupting *papG* can reduce virulence and adhesion. Gupta et al. [72] reported that by using CRISPR-based nanocomposites to target the *papG* gene in *E. coli*, PapG expression can be significantly reduced, and pathogenicity is weakened in model organisms. This suggests that targeting PapG may serve as an effective antivirulence strategy.

### HlyA

RTX proteins are produced by various Gram-negative bacteria and are endowed with multiple functions, ranging from bacterial adhesion and biofilm formation to the activities of bacteriocins, lipases, and proteases [78,79]. Many RTX proteins are pore-forming toxins that have the ability to disrupt host cell membranes. The prototype RTX pore-forming toxin, HlyA, is secreted by pathogenic variants of *E. coli* and is one of the most extensively studied exotoxins in its class [80]. *E. coli* of HlyA is a pore-forming toxin with a molecular weight of 110 kDa, primarily secreted to the extracellular environment via the type I secretion system (T1SS), spanning both the inner and outer bacterial membranes, and is a key virulence factor [81–83]. The T1SS of Gram-negative bacteria can secrete various proteases, lipases, S-layer proteins, and toxins, with the T1SS of HlyA being a typical representative [84]. The N-terminal hydrophobic region of HlyA (amino acid residues 238–410) plays a key role in its pore-forming and hemolytic activity in biological and artificial membranes [85]. In addition, the activity and stability of HlyA depend on its complex formation with LPS through hydrophobic interactions, and the binding of LPS significantly enhances the biological function of this toxin [86]. HlyA causes the efflux of metabolites such as Ca^2+^, K^+^, and ATP by forming pores in the membranes of its target cells, and depending on the concentration and duration of action, it can induce cell lysis, necrosis, or apoptosis [80,87,88] (Figure 3). Verma et al. [89] found that HlyA could induce about 24% cytotoxicity in THP-1 macrophages after 2 hours of stimulation. In the pathogenesis of endometritis, HlyA, on one hand, promotes bacterial invasion into the deeper uterine tissues through its cytotoxic effects on epithelial and stromal cells, and on the other hand, suppresses early innate immune responses by delaying neutrophil infiltration. At the same time, HlyA-mediated tissue damage can further enhance bacterial survival and invasion abilities, thereby exacerbating the pathological progression of uterine infection [90]. In summary, compared with adhesins that only mediate initial colonization, HlyA significantly enhances the pathogenicity of *E. coli* by inducing tissue damage and weakening the early immune response. HlyA may promote bacterial entry into uterine tissue by damaging endometrial epithelial and stromal cells, while also impairing early innate immune responses in the uterus and potentially delaying neutrophil recruitment [91]. From a therapeutic perspective, HlyA is a potential virulence target in *E. coli*-associated endometritis. Recent studies have shown that specific monoclonal antibodies against *E. coli* α-hemolysin can recognize α-Hly in the culture supernatants of clinical isolates and inhibit its hemolytic activity [92]. Catalytically inactivated HlyA has demonstrated good immunogenicity and protective effects in a mouse ExPEC infection model [93]. These results suggest that antibody neutralization or toxoid vaccines may provide new approaches for the prevention and treatment of *E. coli* endometritis in dairy cows.

### CNF1

CNF1 is a bacterial protein toxin that can enhance the invasiveness of *E. coli* by regulating the activity of Rho GTPases in host cells [94] (Figure 3). CNF1 binds to host cells and modifies key signaling proteins in the Rho GTPase family, thereby continuously activating these pathways [95–97] (Figure 3). Continuous Rho activation can disrupt epithelial junctions and barrier function, and upregulate inflammatory factors such as IL-6, IL-8, and TNF-α through the NF-κB pathway, inducing neutrophil infiltration, edema, and epithelial damage [98]. The sustained activation of this pathway severely disrupts various fundamental cellular processes, including cytoskeletal dynamics, the cell cycle, transcriptional regulation, survival, and migration [99,100]. Studies have shown that *E. coli* strains expressing CNF1 can induce damage to the endometrial epithelium and promote the secretion of inflammatory cytokines [101]. Although CNF1 is included in the screening range of *E. coli* virulence factors in bovine endometritis, it has not been frequently detected in studies. Therefore, CNF1 is considered an important potential pathogenic factor of *E. coli* causing bovine endometritis.

### LPS

LPS is a major component of the outer membrane of Gram-negative bacterial cell walls and an important endotoxin, playing a key role in bacterial physiological functions and host immune recognition [102] (Figure 3). LPS molecules are structurally composed of three regions: the hydrophobic Lipid A, the core oligosaccharide, and the O-antigen polysaccharide [103–105]. Lipid A confers stability to the outer membrane of Gram-negative bacteria through its highly ordered structure, reducing permeability to lipophilic molecules [106]. The core oligosaccharide structure is relatively conserved and connects lipid A to the O antigen. The O antigen is composed of repeating oligosaccharide units and is located at the outermost layer of the LPS molecule. It is highly variable and serves as the main basis for O serotyping, but not all Gram-negative bacteria carry this structure [107].

As a key virulence factor of pathogenic *E. coli*, LPS is also a typical pathogen-associated molecular pattern and is often used to establish experimental models related to bovine endometritis [76,108,109]. Studies have shown that the survival rate of bovine endometrial epithelial cells decreased to 75.13% after stimulation with 5 μg/mL LPS for 6 hours, and the expression of IL-6, IL-8, IL1β, and TNF-α mRNA increased significantly [110]. Unlike other virulence factors, LPS exerts its virulence mainly by stimulating the host inflammatory response. It can be recognized by host pattern recognition receptors and acts as a core initiator of inflammatory responses, participating in the development of endometritis [16] (Figure 3). Importantly, the inflammatory activity of LPS may vary depending on the strain or serotype of *E. coli* [111,112]. This variability is mainly attributed to the structural heterogeneity of LPS, which may affect host immune recognition and downstream inflammatory responses [113]. Therefore, in *E. coli*-related bovine endometritis, LPS represents a type of endotoxin virulence factor whose main feature is inducing host inflammatory responses [114].

### K1 antigen

The main immunogenic structures on the surface of *E. coli* are LPS and capsular polysaccharides (CPS), corresponding to O antigens and K antigens, respectively, which serve as the basis for host immune recognition and serotyping [115]. Among them, K1 capsular polysaccharide, as a key virulence factor of extraintestinal pathogenic *E. coli*, endows *E. coli* with invasive characteristics. The capsule is composed of α-2,8-linked polysialic acid, forming a highly hydrated long-chain polysaccharide structure [116]. The pathogenic mechanism of K1 capsular polysaccharide lies in its significant resistance to the host’s innate immune effectors. K1 capsular polysaccharide forms a thick, viscous gel-like physical barrier on the outermost layer of the bacterial body, providing the bacteria with physical anti-phagocytic protection [116,117] (Figure 3). In addition, K1 capsular polysaccharide can significantly inhibit the effective deposition of complement components (such as C3b) and antibodies (IgG) on the bacterial surface, enabling it to resist neutrophil phagocytosis [118] (Figure 3). Even if a small number of opsonin molecules adhere, they are limited to the capsular polysaccharide network and cannot effectively bind to the Fc receptors or complement receptors on the surface of neutrophils, thereby blocking opsonophagocytosis, a key antibacterial mechanism. K1 capsule polysaccharides form a protective barrier, synergistically inhibit complement activation, and regulate the phagocytosis process, promoting bacterial survival and colonization [119,120] (Figure 3). *E. coli* carrying the K1 capsule-associated virulence gene kpsMTII is detected at a significantly higher rate in cows with clinical metritis and endometritis than in healthy cows [121]. It indicates that this virulence factor is closely associated with the occurrence of uterine inflammation in cows.

### O antigen

O antigen is the outermost structural component of the LPS of Gram-negative bacteria and serves as the basis for the O serotyping of *E. coli* [122]. O antigen is an important determinant of surface-associated virulence in *E. coli*, not only promoting serotype diversity but also aiding bacterial survival and persistence within the host [123]. O antigen effectively suppresses the host’s immune response by inhibiting epithelial cells from producing cytokines and chemokines, and by resisting phagocytosis and killing by neutrophils and monocytes [124,125] (Figure 3). Secondly, the physical barrier formed by polysaccharide chains can effectively resist the host’s humoral and cellular immune mechanisms, including neutralizing the bactericidal activity of lysozyme, hindering complement deposition, and further defending against phagocytosis [126] (Figure 3). In addition, besides participating in host immune evasion, the O antigen is also an important recognition receptor for various bacteriophages [127]. This structure can nonspecifically block the binding of bacteriophages to *E. coli* surface receptors, thereby further promoting bacterial colonization and survival in the uterus and facilitating the development of endometritis [128]. Therefore, the O antigen not only confers serotype specificity to *E. coli*, but also, through the aforementioned synergistic effects, significantly enhances the bacteria’s ability to survive and persist within the uterus, thereby promoting the establishment and ongoing infection of endometritis. Compared with the K1 antigen, which mainly provides anti-phagocytic and anti-complement protection as a physical capsule barrier, the O antigen plays a broader role in promoting serotype diversity and enhancing resistance to humoral and cellular immune defenses.

### Siderophore

Siderophores are key virulence factors that enable *E. coli* to overcome host nutritional immunity, successfully colonize, and cause bovine endometritis. Although the postpartum uterus contains abundant nutrients, iron-binding proteins such as lactoferrin in the host body fluids chelate free Fe^3+^, creating an extremely iron-deficient environment to inhibit the growth of pathogens. To overcome this “iron blockade”, *E. coli* synthesizes and secretes siderophores with a very high affinity for Fe^3+^ (such as enterobactin). These siderophores can competitively “hijack” iron ions from host iron-binding proteins, forming siderophore-iron complexes [19] (Figure 3). Subsequently, this complex is recognized by highly specific receptor proteins on the bacterial outer membrane and efficiently transported into the cell through a complete system composed of outer membrane TonB-dependent receptors, periplasmic binding proteins, and inner membrane ABC transporters [129–131]. Once sufficient iron is obtained, *E. coli* can utilize it as a key cofactor to drive essential life activities such as respiratory chain energy metabolism, antioxidant stress response, and toxin synthesis, thereby proliferating massively, overcoming the host’s immune defenses, and ultimately leading to the occurrence and progression of disease [132–134]. Studies have shown that in pathogenic *E. coli* isolates from cows with metritis or endometritis, approximately 94% of samples carry the Yersiniabactin receptor gene *fyuA*, indicating that iron acquisition plays a crucial role in uterine infection [121]. In addition to Yersiniabactin, pathogenic *E. coli* typically encode multiple siderophore systems (including aerobactin, enterobactin, and salmochelin), which also aid in iron uptake and the enhancement of bacterial virulence [135,136]. Therefore, efficient iron acquisition significantly enhances the adaptability of uterine bacteria, promotes their persistence under host immune pressure, and drives the occurrence and progression of endometritis [137].

### Biofilm

Biofilms are usually complex structures of microbial communities composed of DNA, proteins, and polysaccharides [20,138] (Figure 3). They play a key role in the virulence of *E. coli* during bovine endometritis [139]. Bacteria enhance their tolerance to antibiotics and their ability to resist the host immune system by producing large amounts of extracellular polymeric substances, mainly polysaccharides [138] (Figure 3). The formation of biofilms begins with bacterial adhesion to the endometrial surface, followed by the accumulation and maturation stages, during which the coordinated expression of related genes promotes the formation of biofilm structures and the establishment of nutrient channels [140]. Notably, virulence-related genes such as *fimH*, *papC*, and *hlyA* are involved in adhesion and the establishment of biofilms, with *fimH* playing a key role in type 1 fimbriae-mediated attachment and pathogenicity [141–143]. Through biofilm formation, pathogenic *E. coli* in the endometrium can survive in an adverse host environment, evade antimicrobial treatment, and promote persistent infection and disease recurrence. Combined with efficient iron acquisition, the formation of biofilms enables pathogenic *E. coli* to survive in the endometrium, resist host defenses, and establish chronic infections [144]. Importantly, biofilm persistence has significant clinical implications. Biofilm-associated *E. coli* exhibit increased tolerance to antimicrobial treatments and host immune responses, which may contribute to prolonged uterine infections, reduced treatment efficacy, recurrent endometritis, and delayed uterine recovery [20,141]. This persistence can negatively affect reproductive performance in dairy cows [1,24].

## *E. coli* pathogenesis in bovine endometritis

The pathogenic process of *E. coli* inducing endometritis in dairy cows is the result determined by multiple virulence factors [44,48]. Based on the virulence factors discussed above, uterine pathogenic *E. coli* strains possess multiple pathogenic traits, including enhanced adhesion, invasion, and inflammation-activating capacities. This suggests that their pathogenic process can be divided into several stages, including adhesion and colonization, immune evasion, inflammatory damage, persistent infection, and reproductive pathological outcomes [76].

## Adhesion and colonization

**Figure 4.**
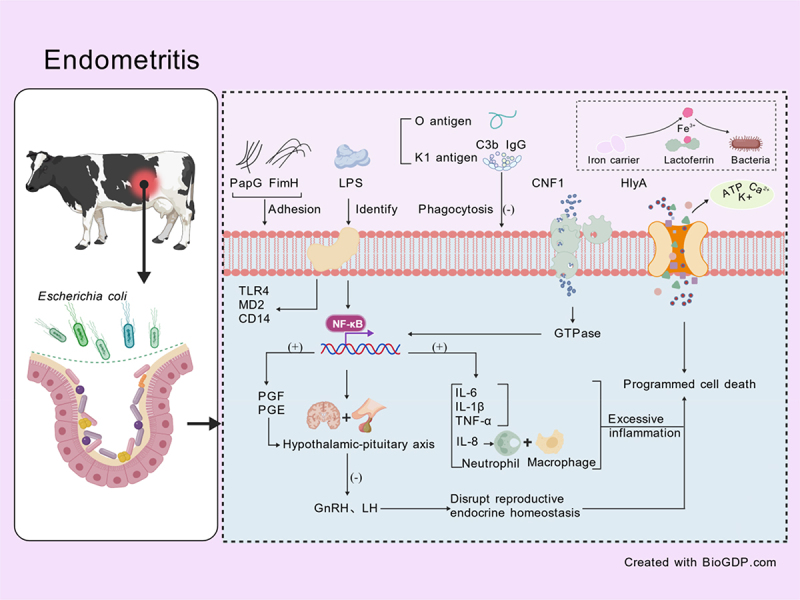
Pathogenic mechanism and pathological progression of *E. coli*-induced endometritis in dairy cows. This figure systematically illustrates the multi-step pathogenic process of *E. coli* in the development of endometritis in dairy cows. It begins with adhesion and colonization: bacteria recognize and bind to receptors on the surface of uterine epithelial cells through adhesins such as FimH and PapG, achieving initial colonization. This is followed by immune evasion: capsular polysaccharides (e.g. K1 antigen) mask pathogen-associated molecular patterns, inhibiting phagocytosis and complement activation to escape host immune clearance. Next, it induces tissue damage and nutrient acquisition: HlyA forms transmembrane pores, causing cell lysis and necrosis; CNF1 activates Rho GTPases, disrupting the cytoskeleton and promoting release of inflammatory mediators. Together, these factors exacerbate tissue destruction and release nutrients. Subsequently, it triggers an inflammatory burst: LPS activates pro-inflammatory cytokines (such as IL-1β, IL-6, TNF-α) and chemokines (such as IL-8) through the TLR4/NF-κB signaling pathway, recruiting neutrophils and other immune cells, leading to excessive inflammation. Ultimately, it results in persistent infection and pathological outcomes: bacteria acquire iron through siderophore systems and form biofilms to resist clearance, with ongoing inflammation and tissue damage leading to disruption of uterine endometrial structure and function.

After calving, the postpartum uterus of lactating cows is exposed to microbial invasion through the open cervix and is rich in lochia, creating high susceptibility to bacterial infection [145,146]. In this process, the adhesion and colonization of *E. coli* are the critical first steps in initiating infection, as they effectively prevent the bacteria from being flushed out by uterine peristalsis or secretions [147]. This pathogenic process mainly depends on key virulence factors, especially adhesins, which can bind to host receptors with high affinity and play a central role in bacterial colonization and infection [148] (Figure 4). As described above, type I pili and P pili represent two important adhesive structures of *E. coli* [149]. Their tip adhesins, FimH and PapG, mediate attachment to bovine endometrial epithelial cells through recognition of distinct host receptors [56,68,150–153]. Because these adhesins target different receptor types, they may play complementary rather than redundant roles during early colonization. However, direct evidence for a synergistic interaction between FimH and PapG in bovine endometritis-associated *E. coli* remains limited and requires further investigation. FimH and PapG are thought to play complementary roles during host colonization, with FimH mediating initial adhesion and PapG promoting stable tissue retention [154]. However, whether there is a synergistic interaction between FimH and PapG in bovine endometrial pathogenic *E. coli* requires further investigation.

In addition to mediating initial attachment, pili and their adhesins contribute to the establishment and persistence of infection. Bacterial surface structures can participate in invasion, motility, and host pro-inflammatory activation, while surface polysaccharides and adhesins may jointly support biofilm formation and bacterial survival under physical, chemical, and biological stress [155–158]. Given the central role of adhesins in tissue tropism, targeted adhesion, biofilm formation, antibiotic resistance, and bacterial internalization [159–161], they are considered promising targets for antimicrobial development [162]. Therefore, blocking adhesin function may represent a potential strategy for preventing bacterial infections such as bovine endometritis.

## Immune evasion

Macrophages and conventional dendritic cells continuously monitor mucosal interfaces and identify invading pathogens. However, pathogenic *E. coli* actively evades immune clearance using various virulence factors, which is particularly prominent in the process of causing bovine endometritis. The remarkable versatility of *E. coli* is reflected in the diversity of surface polysaccharides across different strains [163] (Figure 4). Capsular polysaccharides are crucial for bacterial virulence because they are involved in important biological processes, including adhesion and resistance to the host’s immune responses [164,165]. During infection, K1-type capsular polysaccharides mainly reduce immune recognition, weaken complement and antibody-mediated opsonization, and limit effective clearance by phagocytes, while O antigens further enhance resistance to humoral and cellular immunity [126,166,167]. These immune evasion effects allow *E. coli* to maintain local colonization even when the host has already initiated an inflammatory response, and create conditions for subsequent tissue damage, inflammation amplification, and persistent infection. Furthermore, the persistence of uterine infection is profoundly influenced by the modulation of local adaptive immune mechanisms. Recent evidence indicates that during endometritis, the microenvironment can lead to marked dysfunction and altered tissue residency patterns of CD4^+^ and CD8^+^ T cells in the endometrium [168]. This impairment of T cell-mediated adaptive immunity further dampens the host’s ability to clear the invading pathogens, effectively synergizing with bacterial surface evasion strategies. In addition to local immune-pathogen interactions, recent advances have also highlighted the important role of systemic microbiome-host interactions. Evidence shows that dysbiosis of the microbial community originating from local infections has profound systemic effects on overall host physiology and metabolic homeostasis [169]. Therefore, to study the pathogenic mechanism of endometritis, it is necessary to consider it comprehensively from the perspective of microecology and overall physiological state.

## Tissue injury

During the development of bovine endometritis, pathogenic *E. coli* secretes multiple toxins, forming a multi-step, synergistic pathogenic network. HlyA, as a pore-forming toxin, is released from the bacterial cytoplasm into the host environment and directly attacks uterine epithelial cells [82,170,171]. HlyA inserts into the lipid bilayer, forming hydrophilic transmembrane pores approximately 2 nanometers in diameter, severely compromising cell membrane integrity, ultimately leading to cell lysis due to high-density pore formation [87,172] (Figure 4). In exerting its toxicity, HlyA first binds to the host cell membrane in a calcium-dependent electrostatic manner as a monomer and, with the assistance of surface receptors, enhances pore formation efficiency [173–175]. HlyA can lyse various mammalian cells, including red blood cells, epithelial cells, and leukocytes, creating conditions for further bacterial colonization and invasion [176,177]. During infection, CNF1 binds epithelial cell surfaces, enters via receptor-mediated endocytosis, traffics through endosomes, and releases its catalytic domain into the cytoplasm [178]. Once inside the cell, CNF1 primarily targets Rho GTPases, disrupting the precise regulation of the actin cytoskeleton and leading to cellular structural disarray [179,180]. Damage to epithelial cells rapidly activates innate immune responses. CNF1 plays the role of an “inflammation amplifier” in this context. It promotes NF-κB signaling, inducing the production of pro-inflammatory cytokines (IL-6, IL-8, and TNF-α) and COX-2 expression, thereby promoting neutrophil recruitment to the site of infection [97,181,182]. However, HlyA acts as an “immune disruptor.” It exhibits potent toxicity against recruited neutrophils and other immune cells, directly inducing lysis, necrosis, or apoptosis through pore formation, thereby weakening the body’s first line of defense against bacteria [87]. In addition to inducing neutrophil apoptosis and necrosis, it can also trigger GM-CSF-mediated accumulation of M1 macrophages, significantly enhancing local uterine immunopathological damage and inflammation [83,87]. The progression of the disease is often the result of the combined action of multiple toxins.

## Inflammatory outbreak

In the pathogenesis of bovine endometritis, bovine endometrial epithelial cells (BEECs), as the first line of physical defense, are the first to come into contact with pathogens [183,184]. Its pathogenic process begins when the bacteria reach the infection site using flagella-mediated motility (identified by H serogroup) and attach to the endometrial epithelial cells through adhesins [185,186]. Subsequently, LPS (identified by the O serogroup) serves as the main toxin component, initiating the host immune response [187]. LPS can be recognized by a receptor complex composed of TLR4, CD14, and MD2, and mediate the transmission of inflammatory signals into the cell through TLR4 [188,189]. The innate immune system of mammals recognizes pathogen-associated molecular patterns through pattern recognition receptors, and the Toll-like receptor family is the most important class of pattern recognition receptors [190]. Studies have shown that the expression of TLR4 is significantly upregulated during the infection process [191]. After recognition, it mediates the transmission of inflammatory signals to the cell and activates the downstream nuclear factor-κB (NF-κB) signaling pathway [192,193]. As a key transcription factor, NF-κB rapidly initiates the gene transcription and release of various inflammatory mediators, including cytokines (IL-1β, IL-6, TNF-α), chemokines (especially IL-8), as well as interferons and prostaglandins [11,194]. LPS stimulation regulates local endocrine function by secreting prostaglandin F_2__α_ (PGF) and prostaglandin E_2_ (PGE) [16]. Studies have shown that endometrial cells attacked by LPS preferentially secrete PGE [16,195]. These inflammatory mediators, with IL-8 as a potent chemotactic factor, can recruit inflammatory cells such as neutrophils and macrophages to the site of infection, working together to eliminate pathogens [11,196]. An appropriate inflammatory response helps eliminate pathogens, but if the response is excessive or prolonged, it can lead to cell apoptosis and endometrial damage. Even subclinical endometritis, characterized by increased polymorphonuclear leukocytes and persistent low-grade inflammation despite the absence of obvious clinical signs, can impair the uterine environment, reduce conception rates, and prolong the time to pregnancy [197]. In addition to directly activating inflammatory pathways, LPS can also exacerbate pathological processes through epigenetic mechanisms. Studies have shown that LPS can alter the DNA methylation patterns of endometrial cells, thereby disrupting immune balance and cell adhesion processes [198]. In addition, the effects of LPS go far beyond the local uterus; it can also inhibit the hypothalamic-pituitary axis, reduce the secretion of GnRH and LH, lead to decreased levels of estradiol and progesterone in peripheral blood, disrupt overall reproductive endocrine homeostasis, and ultimately prolong the interval between calvings [12,195,199]. It is noteworthy that periparturient dairy cows are often in a state of negative energy balance [200]. Their reduced feed intake and elevated levels of non-esterified fatty acids in the blood can initially impair neutrophil function, significantly increasing susceptibility to LPS attacks, thereby making them more prone to developing severe endometritis [201]. Therefore, precisely regulating the activation intensity of the TLR4/NF-κB signaling pathway is crucial for controlling excessive inflammatory responses, reducing tissue damage, and maintaining reproductive function.

## Persistent infection

During the development of bovine endometritis, pathogenic *E. coli* effectively overcomes host defenses and establishes persistent infections through two key strategies: a sophisticated iron acquisition system and biofilm formation (Figure 4). First, to cope with the extreme iron-deficient environment caused by postpartum uterine lactoferrin and other factors, *E. coli* actively secretes siderophores, such as enterobactin, which have a very high affinity for Fe^3+^ [202]. These siderophores can competitively seize iron ions from host iron-binding proteins and then transport the iron into the cell through outer membrane-specific receptors (such as FepA) and TonB-dependent transport systems [19,129,131]. This efficient system not only provides the essential nutrients for bacterial proliferation but also significantly enhances its virulence expression, forming the basis for its survival and pathogenicity in nutrient-limited environments.

After successfully acquiring iron and achieving initial proliferation, the bacteria further initiate another key adaptive strategy-the formation of biofilms [203]. The process begins with the initial attachment to the surface of endometrial epithelial cells mediated by adhesins such asFimH [149]. Subsequently, the bacteria construct a biofilm matrix with a three-dimensional structure by secreting extracellular polymeric substances (EPS) [204]. This matrix not only blocks antibiotic penetration and immune cell attacks, but also facilitates nutrient exchange and waste removal through its internal water channels, thereby maintaining bacterial viability and stability [20,138]. In summary, active iron metabolism mechanisms provide pathogenic *E. coli* with the iron essential for survival and proliferation, while biofilm formation confers strong physical protection and a population survival advantage. The synergy between the two enhances bacterial survival in the harsh intrauterine environment. It also increases resistance to host clearance mechanisms and antimicrobial treatments. As a result, persistent infection and clinical recurrence may occur, making disease prevention and control more challenging.

## Pathological outcome

Pathogens like *E. coli* can trigger the innate immune response in the cow’s uterine lining. Too much inflammation can damage the endometrial structure and function, which in turn promotes the onset and development of endometritis in cows [205,206]. The process is characterized by uncontrolled persistent inflammation, accompanied by local microcirculatory disturbances, leading to tissue ischemia, hypoxia, and even necrosis [198,207]. As inflammatory exudate, necrotic tissue, and a large number of infiltrating inflammatory cells accumulate in the uterine cavity, affected cows often exhibit clinical symptoms such as purulent or mucopurulent vaginal discharge, uterine enlargement, and delayed uterine involution [208]. In addition, complications during parturition, such as retained placenta, difficult labor, twin pregnancies, and stillbirths, can significantly increase the risk of endometritis [209].

A long-lasting inflammatory environment not only directly damages the uterine lining but also seriously disrupts reproductive hormone function and affects embryo implantation [210,211]. Therefore, the condition often shows up as reproductive problems, like significantly lower conception rates and higher early embryo loss, putting ongoing economic pressure and production challenges on the dairy industry [1,212].

## Therapeutic methods

**Figure 5.**
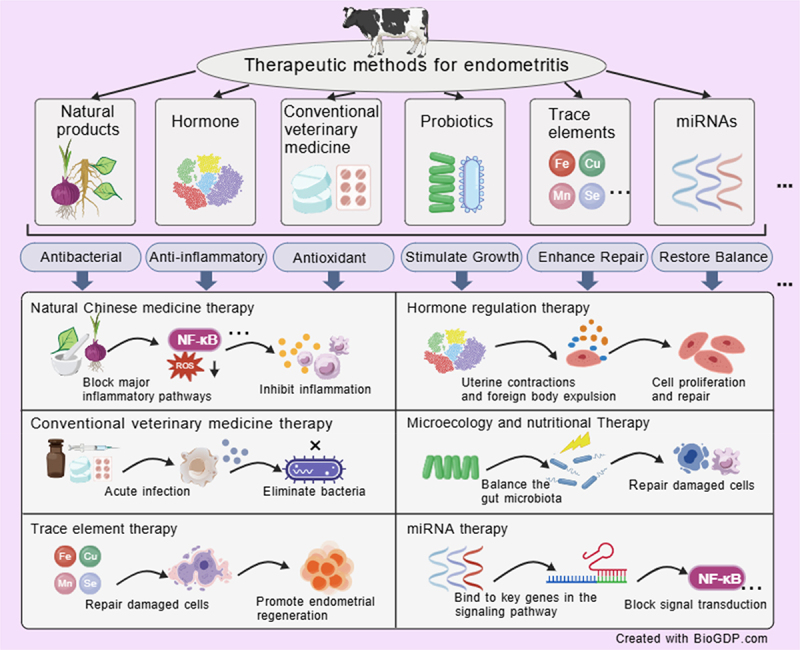
This figure systematically illustrates six major therapeutic strategies for endometritis, encompassing natural products (including herbal medicine and polyphenolic compounds), hormonal therapy, conventional veterinary medicine, probiotics, trace elements, and miRNA-based therapy. Their therapeutic effects involve multiple shared mechanisms, including anti-inflammatory, antibacterial, and antioxidant actions, as well as the promotion of tissue repair and proliferation, and regulation of the microbiota. In clinical application, these strategies have distinct emphases, respectively targeting key aspects such as acute infection control, inhibition of inflammatory pathways, tissue and cell repair, and modulation of the immune microenvironment.

The treatment strategies for bovine endometritis are continuously being optimized and innovated. Current treatment methods, including natural product therapy, conventional veterinary medicine, hormone regulation, probiotic intervention, and trace element supplementation, have shown significant efficacy (Figure 5). However, these emerging therapies are still in the developmental stage and require further mechanistic research and rigorous clinical trials to comprehensively assess their safety and effectiveness for clinical application.

### Natural products

#### Traditional Chinese medicine and its preparations

Traditional Chinese medicine, such as herbal medicine and its formulations demonstrate unique value in the treatment of bovine endometritis due to their holistic regulatory advantages with multiple components and multiple targets. Clinically, compound herbal preparations show broad prospects because of their synergistic effects. For example, Yimu Qinggong San achieves multi-target anti-inflammatory effects by inhibiting the NF-κB signaling pathway and regulating M2 macrophage polarization [213]; German herbal veterinary medicine EucaComp® PlantaVet (containing ingredients such as Calendula officinalis L., Mellissa officinalis L., Origanum majorana L. and Eucalyptus globulus Labill) has anti-inflammatory, antibacterial, and antioxidant effects, with clinical efficacy comparable to the conventional antibiotic cephapirin [22]. Single herbs also have distinctive features. Danshen (Botanical name: Salvia miltiorrhiza), a medicine that promotes blood circulation and removes blood stasis, can improve microcirculation, regulate energy metabolism, and suppress inflammatory responses [214–216]. Studies have shown that Salvia miltiorrhiza significantly alleviates clinical symptoms of endometritis by reducing uterine inflammation and improving tissue congestion [217].

In terms of standardization and modern formulation development, TCM extracts have unique advantages due to their clearly defined composition and controllable quality (Figure 5). Infusing 15% garlic extract into the uterus can significantly reduce bacterial load and boost local immune response, which can improve cure rates and conception rates, suggesting it has good potential for clinical use [218]. Eucalyptus robusta leaves methanolic extract shows antibacterial effects comparable to cephalosporins in experimental rat endometritis models; however, its efficacy in clinical bovine endometritis still requires further investigation [219]. Of particular note are plant essential oil components, such as cinnamon oil and clove oil, which, due to their lipophilic, small-molecule characteristics and excellent tissue penetration, exhibit significant inhibitory effects against pathogens like pyogenic actinomycetes [220]. The latest evidence further supports the antibacterial potential of plant-derived essential oils against bovine pathogens. Mustafa et al. [221] demonstrated that several plant essential oils exhibited broad-spectrum antibacterial activity against clinically relevant bovine bacterial isolates. These findings highlight their potential as alternative or supplementary therapies for managing cattle diseases. They lay a solid foundation for the development of novel transdermal formulations.

In-depth mechanistic studies reveal that active components of traditional Chinese medicine exert therapeutic effects by precisely regulating key signaling pathways. In terms of inflammation regulation, andrograpanin, catalpol and leonurine can specifically block TLR4-mediated NF-κB signaling, inhibiting the expression of pro-inflammatory factors such as IL-1β, IL-6, and TNF-α [109,222,223]; matrine, on the other hand, protects against *Staphylococcus aureus* (*S. aureus*) infection by dose-dependently inhibiting the TLR2/NF-κB pathway [224]. Regarding oxidative stress regulation, andrographolide and berberine upregulate the expression of antioxidant enzymes such as HO-1 by activating the Nrf2 signaling pathway, effectively reducing oxidative stress levels and suppressing inflammatory responses [225–227]. Notably, tanshinone IIA can synergistically inhibit NF-κB while activating the Nrf2 pathway, forming a dual anti-inflammatory and antioxidant enhancement mechanism [228,229]. Additionally, other active ingredients act through different pathways. Ginsenoside Rg1 alleviates endometrial epithelial-mesenchymal transition and fibrosis by inhibiting the reactive oxygen species-NLRP3 (ROS-NLRP3) pathway, with efficacy confirmed in animal experiments [230]. Honokiol effectively mitigates LPS-induced endometrial inflammation and cell apoptosis by regulating endoplasmic reticulum stress signaling pathways [231]. Similarly, astaxanthin not only activates the endometrial defense barrier but also inhibits oxidative stress and cell apoptosis, while promoting cell viability and growth factor production [232]. Recent evidence suggests that medicinal plants may enhance fertility by regulating reproductive hormones, alleviating oxidative stress, and improving ovarian and oocyte function [233]. These findings provide additional mechanistic support for plant-based fertility regulation interventions. In summary, traditional Chinese medicine has established a multidimensional therapeutic system encompassing anti-inflammatory, antibacterial, antioxidant, and tissue repair effects, providing a comprehensive and effective solution for the clinical prevention and treatment of bovine endometritis.

#### Polyphenolic compounds

Polyphenolic compounds are a class of naturally occurring compounds widely found in plants, and they have attracted much attention due to their various bioactivities, such as antioxidant and anti-inflammatory effects. Polyphenolic components alleviate tissue damage caused by oxidative stress through different pathways [234]. Studies have shown that intake of polyphenolic substances can reduce and prevent the risk of existing disease symptoms [235]. For example, cyanidin-3-O-glucoside (C3G) specifically inhibits myeloperoxidase (MPO) activity, lowers oxidative stress markers, and alleviates endometrial oxtion and oxidative stress responses [236]. Regarding the MAPK signaling pathway, isorhamnetin significantly improves the damage of non-esterified fatty acids to endometrial epithelial cells by inhibiting this pathway [237]. Baicalin exerts anti-inflammatory effects by inhibiting the NF-κB and JNK signaling pathways, thereby attenuating inflammatory responses induced by *E. coli* and *S. aureus* infection in endometritis [238]. In addition, ferulic acid, chebulagic acid, and punicalagin alleviate LPS-induced inflammatory injury in bovine endometrial epithelial cells primarily through suppression of the NF-κB and MAPK signaling pathways, suggesting their potential as promising therapeutic candidates for the treatment of *E. coli*-associated bovine endometritis [239–241]. Furthermore, polyphenolic compounds exert fine regulation at the level of inflammatory mediators. Epigallocatechin gallate (EGCG) protects the mouse endometrium by activating SIRT1 to reduce oxidative stress and apoptosis [242]. Hydroxytyrosol effectively scavenges the excess ROS accumulation induced by LPS stimulation and activates the Nrf2 signaling pathway, enhancing the function of the endogenous antioxidant defense system [207]. While naringin alleviates oxidative stress-induced autophagic damage and apoptosis by regulating key nodes of endoplasmic reticulum stress and inhibiting the PI3K/AKT signaling pathway [243]. Curcumin helps improve the inflammatory environment in the uterus by blocking LPS-induced inflammation, reducing PGE_2_ and pro-inflammatory cytokines [244,245]. Ullah et al. [246] reported that essential oils and extracts from aromatic plants have antibacterial, anti-inflammatory, and antioxidant effects. These regulatory effects can not only directly alleviate inflammatory damage in the endometrium but also break the vicious cycle of “inflammation-oxidative stress,” creating a microenvironment conducive to tissue repair.

### Hormone

Hormones play an important role in regulating endocrine function as well as immune responses related to uterine health and reproductive performance. Yang et al [247] reported that immune intervention strategies can effectively modulate inflammation and immune processes, thereby promoting improvements in reproductive health and fertility. Prostaglandins (PGs) play a dual regulatory role in the occurrence, development, and outcome of endometritis in cows (Figure 5). When the cow’s endometrium is infected, the expression of prostaglandin D_2_ (PGD_2_) is upregulated [248]. However, PGD_2_ exerts a negative feedback regulatory effect through its receptor DP1. And it inhibits the secretion of inflammatory cytokines, including IL-1β, IL-6, and TNF-α, and downregulates the phosphorylation of p38, ERK, and NF-κB, thereby alleviating endometrial damage [248]. Prostaglandin F_2__α_ (PGF_2__α_) is essential for uterine involution; it promotes uterine smooth muscle contraction and lochia discharge, thereby aiding uterine recovery [249]. Clinical studies have shown that weekly injections of PGF_2__α_ can reduce the incidence of endometritis and improve reproductive performance [250]. The role of prostaglandin E_2_ (PGE_2_) in the regulation of endometrial inflammation and repair is also well understood. Excess PGE_2_ can occur in various inflammatory diseases, including endometritis. Under pathological conditions, *E. coli* or LPS infection of the endometrium induces excessive accumulation of PGE_2_, exacerbating inflammatory responses and tissue damage [251,252]. Its mechanisms can be roughly summarized into the following three types. Activation of the TLR2/4-MyD88/p38 MAPK pathway promotes the expression of PGE_2_ and its synthesizing enzymes (COX-2 and mPGES-1), leading to increased production and pathological accumulation of PGE_2_ [253]. This will increase the expression of pro-inflammatory factors, which in turn worsens tissue damage [254]. PGE_2_ activates PKA via the EP4 receptor, further amplifying the inflammatory response and causing pathological damage to the endometrium [253]. Inhibiting COX-2, mPGES-1, EP4, or PKA can significantly reduce the pathological accumulation of PGE_2_, thereby alleviating inflammatory damage, suggesting that these molecules could serve as therapeutic targets. PGE_2_ upregulates damage-associated molecular patterns (DAMPs), exacerbating the inflammatory cascade [255]. Under certain conditions, such as in bovine endometrial epithelial cells stimulated by LPS, it can downregulate the inflammatory response through the TLR4-NF-κB pathway, exhibiting a certain anti-inflammatory effect, but this effect is influenced by the microenvironment [256]. In addition, PGE_2_ functions similar in infections caused by different pathogens. During *E. coli* infection, PGE_2_ primarily promotes inflammation, exacerbating tissue damage through the TLR2/4-MyD88/p38 MAPK and EP4-PKA pathways; whereas during *S. aureus* infection, it also helps *S. aureus* stick to and invade bovine endometrial epithelial cells [257]. This difference may be related to the PAMPs (pathogen-associated molecular patterns), host receptors, and the mode of activation of downstream signaling pathways.

Estradiol (E_2_) is an endogenous steroid hormone that has been shown to have anti-inflammatory properties [258]. Studies have found that E_2_ significantly reduces the expression of IL-1β, IL-6, TLR4, and NF-κB in a uterine endometritis model induced by *E. coli* [259]. At the same time, E_2_ also promotes endometrial cell proliferation by activating the Wnt/β-catenin pathway, synergistically aiding tissue damage repair [259]. Fang et al. [260] found that E_2_ can ease fibrosis in cow endometrial epithelial cells and suggests this might be related to the GPER-mediated TGFBR3/Smad2/3 signaling pathway. This mechanism significantly reduces the expression of fibrosis markers, thereby maintaining the plasticity of endometrial tissue.

Cortisol, as a representative molecule of glucocorticoids, exhibits complex biphasic effects in regulating inflammation of the bovine endometrium. Studies have shown that cortisol can significantly reduce the inflammatory response of bovine endometrial epithelial cells activated by LPS or heat-inactivated *E. coli* by inhibiting the NF-κB and MAPK signaling pathways [261,262]. This mechanism demonstrates clear anti-inflammatory potential in an LPS-induced acute endometritis model, suggesting that cortisol could be used to control excessive inflammation mediated by bacterial toxins. However, the effects of cortisol are significantly pathogen-dependent. Cui et al. [262] found that in bovine endometrial epithelial cells stimulated with live *E. coli*, cortisol exerted pro-inflammatory effects by enhancing the expression of several pro-inflammatory genes, particularly IL-1β, IL-6, and TNF-α, which contributed to an intensified inflammatory response and increased tissue injury.

Dihydrotestosterone (DHT), as an active metabolite of testosterone, also plays a role in regulating the inflammatory response of bovine endometritis. Studies have found that DHT can significantly inhibit the RPS (pathogen-associated molecular pattern)-activated TLR4/MyD88 signaling pathway in an androgen receptor (AR)-dependent manner, thereby reducing the inflammatory response of bovine endometrial epithelial cells [263]. Notably, this anti-inflammatory effect of DHT is clearly AR-dependent.

Melatonin can inhibit inflammation caused by LPS. It enhances autophagy in endometrial cells to maintain cellular homeostasis and simultaneously inhibits NLRP3 inflammasome activation, reducing IL-1β and IL-18 production [264].

### Conventional veterinary medicine

The drug treatment system for bovine endometritis has evolved into a comprehensive therapeutic regimen that includes antibiotics, non-steroidal anti-inflammatory drugs, and other novel formulations (Figure 5).

In the field of antibiotics, several antimicrobial agents have demonstrated therapeutic potential against bovine endometritis, although their regulatory approval and clinical application may vary across regions. Rifaximin exhibits unique anti-inflammatory properties, exerting an anti-inflammatory effect on stromal endometrial cells by inhibiting the production of IL-6 and IL-8 after LPS stimulation [265]. However, it is primarily of experimental and mechanistic interest and is not routinely approved or used for the treatment of bovine endometritis in some regions. In contrast, β-lactam antibiotics remain among the most widely applied therapeutic options in veterinary practice. Twenty percent oxytetracycline nanoparticles (OTC-NPs) can improve bioavailability and tissue penetration, reduce levels of IL-1, IL-6, and TNF-α, and help repair the endometrium [266]. High-throughput sequencing studies further confirmed that oxytetracycline has specific inhibitory effects on *Bacteroides*, *Trueperella*, *Peptoniphilus*, *Parvimonas*, *Porphyromonas*, and *Fusobacterium* [267]. Cephalosporin antibiotics show differential efficacy in clinical applications. Intrauterine injection of cephapirin benzathine can significantly improve the recovery and reproductive performance of buffaloes with subclinical endometritis [268]. A repeated dosing strategy of cephapirin, with a second infusion 14 days after initial treatment, also enhances reproductive performance and first pregnancy rates in dairy cows [38,269]. In contrast, although ceftiofur can improve the cure rate of endometritis, its effect on enhancing production performance is limited [270]. This difference in efficacy suggests the need to select personalized treatment plans based on clinical symptoms. It should be noted that some uterine-based treatments, such as intrauterine flushing, remain controversial and are not universally considered standard practice. Their acceptance and use vary according to regional veterinary guidelines and local regulations.

In the field of non-antibiotic treatments, nonsteroidal anti-inflammatory drugs demonstrate adjunctive therapeutic value by modulating the inflammatory response [271]. Especially meloxicam, which regulates the proliferation of endometrial epithelial cells under LPS stimulation by interfering with the Wnt/β-catenin and PI3K/AKT signaling pathways [272]. Bromhexine hydrochloride, as an antibacterial adjuvant, exhibits significant antibacterial activity against *E. coli* and *Trueperella pyogenes*, and its synergistic effect with conventional antibiotics provides a feasible approach to reducing antibiotic usage [273]. Furthermore, because the approval status and recommended use of antimicrobial agents differ among countries and regulatory authorities, treatment choices should be interpreted within the context of local veterinary regulations and clinical practice.

### Probiotics

Some probiotics can directly inhibit the growth of common pathogens that cause endometritis (Figure 5). Four probiotic strains, *Lactobacillus rhamnosus*, *Pediococcus acidilactici*, *Lactobacillus sakei*, and *Lactobacillus reuteri*, effectively inhibit *E. coli* infection in vitro [205]. And it suggests that the strength of this inhibitory effect is influenced by the dose of probiotics. Probiotics can alleviate uterine inflammation by regulating key signaling pathways. *Lactobacillus rhamnosus* LGR-1 can inhibit cell membrane damage caused by *Bacillus cereus* toxins and reduce potassium efflux, thereby protecting cell function [274]. At the same time, *Lactobacillus rhamnosus* GR-1 suppresses the activation of NF-κB and MAPKs signaling pathways by inhibiting both MyD88-dependent and independent pathways, thereby inhibiting the release of pro-inflammatory factors [275,276]. In addition, genetically engineered *Lactobacillus johnsonii* expressing GM-CSF reduces *E. coli*-induced inflammation, alleviates uterine tissue damage, and modulates cytokine balance [277]. In the screening of probiotics, *Lactiplantibacillus plantarum* KUGBRC and *Pediococcus pentosaceus* GBRCKU exhibited excellent in vitro probiotic properties, and no virulence genes were detected in their genomes, indicating potential for treating endometritis [278]. Nisin, a natural antimicrobial peptide produced by lactic acid bacteria, has been shown to effectively protect the rat uterus against *S. aureus* infection [279]. This provides a promising strategy for preventing cow endometritis based on probiotics.

### Trace elements

Selenium (Se), as an essential trace element, demonstrates significant biological value in the reproductive health of dairy cows. Recent studies indicate that the multi-pathway regulatory mechanisms of selenium play a key role in the prevention and treatment of bovine endometritis [280] (Figure 5). At the molecular level, selenium significantly activates important signaling pathways such as PI3K/AKT/GSK-3β and Wnt/β-catenin [281]. The mechanism not only enhances the proliferative capacity of bovine endometrial stromal cells but also effectively inhibits apoptosis induced by LPS and cortisol [282,283]. Meanwhile, selenium upregulates the mRNA expression of key growth factors such as *TGFB3* and *VEGFA*, promoting the repair and regeneration of damaged endometrial tissue [282]. In terms of antioxidant defense, selenium regulates the expression of glutathione peroxidases (GPx1 and GPx4), effectively mitigating LPS-induced oxidative stress damage and maintaining endometrial cell homeostasis [284]. Notably, clinical studies have confirmed that subcutaneous injections using a combination of zinc, manganese, copper, and selenium trace elements can significantly reduce the incidence of bovine endometritis and mastitis without affecting reproductive performance or milk production [285]. These findings provide a solid theoretical basis for developing therapeutic strategies based on selenium and other composite trace elements.

### miRNAs

In recent years, an increasing number of studies have shown that microRNAs (miRNAs) play a key role in regulating endometritis (Figure 5). The regulatory mechanisms of miRNAs are diverse, involving multiple key signaling pathways such as Wnt/β-catenin, NF-κB, MAPK, and the NLRP3 inflammasome. For example, miR-92b alleviates inflammation induced by lipoteichoic acid by inhibiting the Wnt/β-catenin signaling pathway [286]. Similarly, miR-204 inhibits the Wnt/β-catenin pathway by targeting *CCND2*, whereas miR-30a reduces oxidative stress and inflammation triggered by LPS or lipoteichoic acid through regulating the MyD88/Nox2/ROS/NF-κB axis [287,288].

In the regulation of the NF-κB signaling pathway, various miRNAs exhibit significant anti-inflammatory effects. The miR-424-5p inhibits NF-κB activation by targeting *IRAK2*, reducing the production of TNF-α, IL-1β, and IL-6 [289]. The miR-505 and let-7c reduce the inflammatory response by inhibiting *HMGB1* and directly blocking NF-κB activation, respectively [290,291]. MicroRNA-211 regulates the expression of *TAB1* in LPS-induced endometritis and inhibits the NF-κB signaling pathway [292]. In addition, miR-19a negatively regulates the NF-κB pathway by targeting *TBK1*, while miR-488 mitigates LPS-induced inflammation by inhibiting the AKT/NF-κB pathway mediated via *Rac1* [293,294].

The regulation of the MAPK signaling pathway is also an important way through which miRNAs exert their effects. The bta-miR-22-3p inhibits the activation of the MAPK pathway by suppressing the *KSR2* gene, while miR-26a suppresses MAPK signaling by inhibiting *MAP3K8*, thereby alleviating LPS-induced endometrial injury [295,296]. In addition, miR-24-3p regulates TLR4/NF-κB signaling and inhibits the release of pro-inflammatory mediators by suppressing the expression of *TRAF6* or *galectin-9* [297,298].

In terms of inflammasome regulation, miR-495 inhibits pyroptosis and inflammatory responses by suppressing the activation of the NLRP3 inflammasome [299], whereas miR-223 exerts a protective effect by limiting NLRP3 activation [300]. In addition, the expression of miR-148a is significantly downregulated in LPS-stimulated endometrial epithelial cells, while its overexpression can effectively alleviate inflammation, suggesting its potential regulatory value [301]. Despite these research findings highlighting the great potential of miRNA in the treatment of endometritis, most miRNA-based approaches are currently still limited to clinical trial studies. So far, no miRNA-based therapeutic product has been routinely approved for use in clinical veterinary practice for the treatment of bovine endometritis [302]. Before widespread application, challenges related to delivery systems, stability, target specificity, safety assessment, and regulatory approval still need to be addressed [303]. Nevertheless, miRNA remains a promising candidate for future diagnostic biomarkers and targeted therapeutic interventions for bovine endometritis.

## Conclusion

Based on the comprehensive review of bovine endometritis, it is concluded that this postpartum uterine disease represents a major challenge to dairy production worldwide, leading to substantial economic losses and impaired reproductive performance. The global prevalence of endometritis exhibits significant spatial heterogeneity, which is influenced by the complex interplay of management practices, environmental conditions, regional pathogen characteristics, and differences in diagnostic methods. *E. coli* is a main but not the only pathogen causing endometritis in cows, and it kicks off infection through a series of carefully arranged virulence mechanisms. Key virulence factors-including FimH and PapG adhesins, HlyA toxin, CNF1, LPS, K1 and O antigens, siderophores, and biofilm-forming capacity, collectively enable *E. coli* to adhere, colonize, evade host immunity, acquire nutrients, induce tissue damage, and trigger persistent inflammatory responses, ultimately leading to endometrial dysfunction and infertility.

Current treatment strategies are increasingly diversified, encompassing antibiotics, hormones, natural products (including traditional Chinese medicine and polyphenols), probiotics, trace elements, and emerging miRNA-based therapies. However, the overreliance on antibiotics has escalated issues of antimicrobial resistance, underscoring the urgency to develop alternative and integrative therapies. Promising approaches such as immunomodulation, microbiota intervention, and multi-target herbal formulations have demonstrated efficacy in modulating key inflammatory pathways like TLR4/NF-κB and oxidative stress responses, offering potential for sustainable and effective disease management.

In summary, combating bovine endometritis requires a holistic understanding of its etiology, pathogen-host interactions, and the dynamics of uterine immunology. Future efforts should focus on integrated control strategies that combine improved herd management, targeted anti-virulence therapies, and immunomodulatory interventions to enhance uterine health, restore fertility, and ensure the economic sustainability of dairy farming.

## Data Availability

The data that support the findings of this study are openly available in Science Data Bank (ScienceDB) at https://doi.org/10.57760/sciencedb.41622, reference number [304].
